# Adenoma detection rates and complications of colonoscopy in patients aged 75 to 79 vs 70 to 74 years: Propensity score-matching study

**DOI:** 10.1055/a-2788-3397

**Published:** 2026-02-03

**Authors:** Osamu Toyoshima, Toshihiro Nishizawa, Shuntaro Yoshida, Tomoharu Yamada, Keisuke Mabuchi, Takuma Kaneko, Mari Mizutani, Hirotoshi Ebinuma, Mitsuhiro Fujishiro, Keisuke Hata

**Affiliations:** 1 577428 Gastroenterology, Toyoshima Endoscopy Clinic, Setagaya, Japan; 2 Department of Gastroenterology and Hepatology, International University of Health and Welfare Narita Hospital, Chiba, Japan; 3 Department of Gastroenterology, Graduate School of Medicine, the University of Tokyo, Tokyo, Japan; 4 38084 Center for Diagnostic and Therapeutic Endoscopy, Keio University, School of Medicine, Shinjuku-ku, Japan

**Keywords:** Endoscopy Lower GI Tract, Polyps / adenomas / ..., CRC screening, Endoscopic resection (polypectomy, ESD, EMRc, ...)

## Abstract

**Background and study aims:**

Several guidelines recommend discontinuation of routine surveillance colonoscopy after age 75 years. Because Japan has one of the longest life expectancies, we considered ceasing at age 80 years. We compared patients aged 75 to 79 years with those aged 70 to 74 years, regarding adenoma detection rate (ADR), mean number of adenomas per colonoscopy, and adverse events.

**Patients and methods:**

This propensity score-matching (PSM) study included patients aged 70 to 79 years with a performance status of 0 to 1 who underwent colonoscopies at Toyoshima Endoscopy Clinic between 2017 and 2024. Patients aged 75 to 79 years were matched with those aged 70 to 74 years for baseline characteristics using the propensity score. ADR, mean number of adenomas per colonoscopy, frequency of respiratory depression, hypotension, and delayed post-polypectomy bleeding were compared between the two groups.

**Results:**

During the study period, 3415 patients were included. The ADR in patients aged 75 to 79 years was higher than that in patients aged 70 to 74 years (66.5% vs 62.2%, *P* = 0.021). Mean number of adenomas per colonoscopy in patients aged 75 to 79 years was higher than that in patients aged 70 to 74 years (1.54 vs 1.38, *P* = 0.014). The two groups did not show significant differences in respiratory depression (2.6% vs 2.3%), hypotension (0.8% vs 1.0%) or delayed post-polypectomy bleeding (0.2% vs 0.4%).

**Conclusions:**

Colonoscopies for patients aged 75 to 79 are safe and effective in Japan.

## Introduction

Colorectal cancer (CRC) is a fatal disease that occurs worldwide. Because CRCs mainly develop from conventional adenomas or serrated polyps, their removal prevents CRC 1 2 . Surveillance colonoscopy has proven beneficial through decreased incidence and mortality of CRC 3 . Endoscopists with high adenoma detection rates (ADRs) could enhance these benefits 4 5 . In addition, there is a controversy regarding when to discontinue surveillance, especially in an aging population. United States guidelines recommend discontinuing routine surveillance colonoscopy after age 75 years 6 , whereas European guidelines suggest stopping endoscopic surveillance at age 80 years 7 . Although the life expectancy is 78.8 years in the United States, Japan has one of the longest at 84.4 years 8 . In Japan, life expectancy is approximately 5 years longer; therefore, we considered ceasing at age 80 years. With advances in endoscopic techniques and equipment, colonoscopies may become safe and effective, even in patients aged 75 to 79 years. In this study, we compared patients aged 75 to 79 years with those aged 70 to 74 years regarding ADR,% mean number of adenomas per colonoscopy (APC), and adverse events (AEs).

## Patients and methods

### Study overview

This retrospective, single-center, propensity score-matching (PSM) study was conducted at the Toyoshima Endoscopy Clinic, a representative outpatient clinic specializing in endoscopy in Japan. Patients aged 70 to 79 years who underwent colonoscopy at our clinic between April 2017 and April 2024 were eligible for the study. Indications for colonoscopy included symptom examination, screening, and surveillance of colorectal polyps. Symptoms included hematochezia, abnormal bowel habits, and abdominal pain. Patients with poor bowel preparation, prior colorectal surgical resection, incomplete cecal intubation, or treatment purpose were excluded. Treatments included planned polypectomy and emergency hematemesis. Colonoscopy was scheduled for patients with a performance status (PS) of 0 or 1 9 10 and American Society of Anesthesiologists (ASA) physical status classification of I or II 11 . PS was divided into five levels ranging from 0 to 4. PS 0, normal activity; PS 1, some symptoms, but still nearly fully ambulatory; PS 2, less than 50%; PS 3, more than 50% of daytime in bed; and PS 4, completely bedridden. ASA physical status classification was divided into six levels ranging from 1 to 6. ASA I, healthy patients; ASA II, mild systemic disease; ASA III, severe systemic disease; ASA IV, constant threat to life; ASA V, moribund patients; and ASA VI, brain-dead patients.

This retrospective study was approved by the Certified Institutional Review Board of the Yoyogi Mental Clinic on July 16, 2021 (approval no. RKK227). We published the study protocol on our clinic website ( www.ichou.com ); thus, patients could opt out if desired. Written informed consent was obtained from all participants. All the clinical investigations were conducted in accordance with the ethical guidelines of the Declaration of Helsinki.

### Colonoscopy

The endoscopy system used was either Olympus EVIS LUSERA ELITE (CV-290) or EVIS X1 (CV-1500). CF-EZ1500D, CF-XZ1200, CF-HQ290, CF-HQ290Z, CF-H290EC, PCF-H290Z, or PCF-PQ260 (Olympus Corp., Japan) were used. Sedation was performed based on patient willingness. Midazolam, pethidine, and/or propofol were used 12 . Pan-colonic chromoendoscopy with indigo carmine was used routinely 13 . Moreover, endoscopic observation was performed using texture and color enhancement imaging, as well as white-light imaging, to increase polyp detection 14 15 . Endoscopic resection techniques included endoscopic mucosal resection and hot or cold polypectomy using snares or forceps 16 17 . Bowel preparations were classified into four groups. We used the Harefield Cleansing Scale because it is the standard scoring method in our institution and is integrated into our reporting system. Grade A was defined as cleanliness or a minor amount of fluid in all colonic segments (good). Grade B was defined as residual semi-solid stool that could be easily removed (average). Grade C was defined as partially removable stool that prevented complete visualization of the mucosa (marginal). Grade D was defined as remaining solid stool that prevented examination (poor) 18 19 . Grade D patients were excluded because of poor patient preparation. Withdrawal time included time required for polypectomy. A withdrawal time of less than 6 minutes was excluded as an inappropriate examination 20 .

Respiratory depression was defined as reduction in oxygen saturation < 90% for > 20 seconds or implementation of oxygen inhalation based on the judgment of the on-site endoscopist 21 . Hypotension was defined as reduction in systolic blood pressure < 80 mm Hg 22 . Delayed post-polypectomy bleeding (DPPB) was defined as bleeding within 14 days of polypectomy that required emergent endoscopy 23 .

### Data collection and outcome parameters

Our electronic endoscopy reporting system was the T-File System (STS Medic, Japan) integrated into the electronic medical record system, the Qualis (BML, Japan). The endoscopy reporting system outputted the information for this study in Microsoft Excel file format 24 25 .

Background information included patient age, sex, indications for colonoscopy, endoscopist, endoscopy system, colonoscope, bowel preparation, withdrawal time, and doses of midazolam, pethidine, and propofol. The indications were divided into three groups: A, evaluation of symptoms; B, screening; and C, surveillance 26 . Endoscopists were classified into two groups: experts and standard 27 . Expert endoscopists were defined as those with > 20,000 colonoscopies. Endoscopy systems were classified into two groups: X1 and ELITE. Colonoscopes were classified into three groups: A, CF-EZ1500D and CF-XZ1200; B, CF-HQ290, CF-HQ290Z, CF-H290EC, and PCF-H290Z; and C, PCF-PQ260 28 .

Outcome parameters were ADR; APC; advanced ADR; mean number of advanced APCs; adenocarcinoma detection rate; sessile serrated lesion detection rate (SSLDR); mean number of sessile serrated lesions per colonoscopy (SSLPC); and frequency of respiratory depression, hypotension, and DPPB. Advanced adenomas included adenomas ≥ 10 mm in size, villous adenomas, and adenomas with high-grade dysplasia.

### Statistical analysis

PSM was used to adjust patient characteristics to reduce effects of selection bias and potential confounding factors. The adjustment items included all baseline patient characteristics such as sex, indication, endoscopist, endoscopy system, colonoscope, bowel preparation, withdrawal time, and doses of midazolam, pethidine, and propofol. Patients aged 75 to 79 years were identified and the propensity score was matched with those aged 70 to 74 years. Matching was performed with a 1:1 matching protocol using nearest-neighbor matching without replacement and a caliper width of 0.1 of the pooled standard deviation of the logit of the propensity score 29 .

After PSM, we analyzed differences in ADR, APC, advanced ADR, advanced APC, adenocarcinoma detection rate, SSLDR, and SSLPC, as well as frequency of respiratory depression, hypotension, and DPPB between the two groups. We performed additional subanalyses by indications for colonoscopy. *P* values for baseline characteristics were calculated using Brunner-Munzel and Wilcoxon signed-rank sum tests for before and after matching, respectively. We assessed *P* values for outcomes using the Wald test with logistic regression. Statistical significance was set at *P* < 0.05. To assess quality of matching, we evaluated covariate balance using standardized mean differences (SMDs), with an SMD < 0.10 considered indicative of adequate balance. In addition, Love plots were generated to visually compare covariate balance before and after matching. Calculations were performed using Bell Curve for Excel version 4.07 (Social Survey Research Information Co., Ltd., Japan).

## Results

**Fig. 1:**
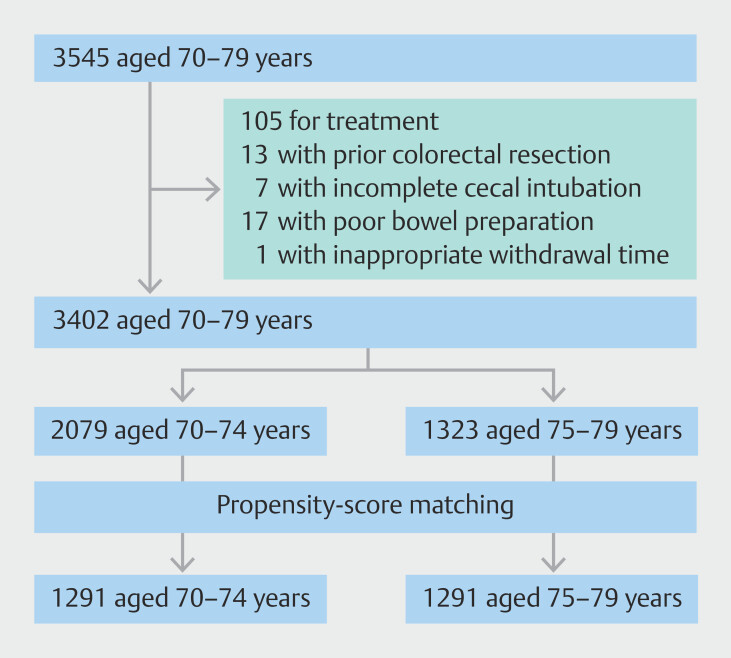
Patient flowchart.

**Fig. 2:**
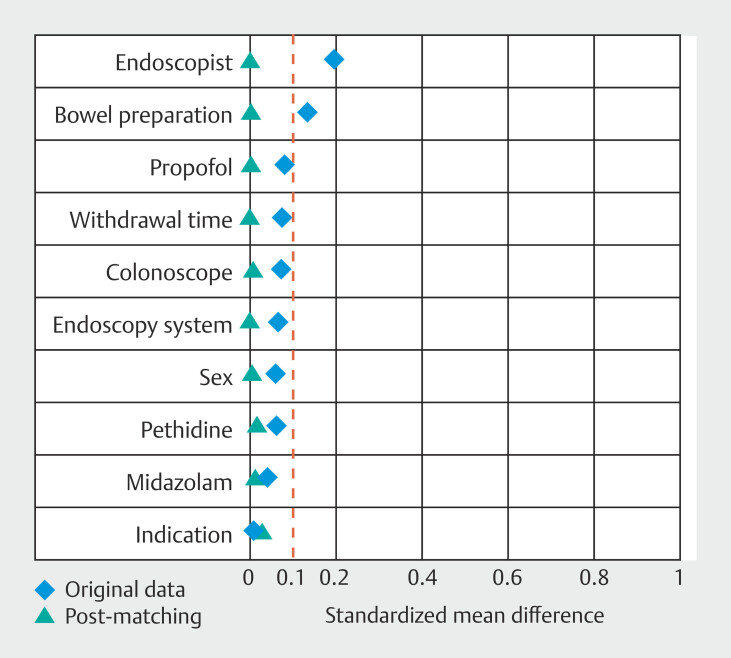
Love plot of the standardized mean difference before and after propensity-score matching. Dashed vertical lines indicate the threshold for acceptable imbalance, defined as an absolute standardized mean difference of 0.10.

During the study period, 3545 consecutive patients aged 70 to 79 years who underwent colonoscopy were enrolled. We excluded 105 patients for therapeutic purposes, 17 patients for poor bowel preparation, 13 patients for prior colorectal resection, seven patients for incomplete cecal intubation, and one patient for an inappropriate withdrawal time. Of the 17 patients with poor bowel preparation, 13 were aged 70 to 74 years and four were aged 75 to 79 years. Of the seven patients with incomplete cecal intubation, four were aged 70 to 74 years and three were aged 75 to 79 years. Finally, 3402 patients were included in the study. There were 2079 patients aged 70 to 74 years and 1323 aged 75 to 79 years. The two age groups were paired and 1,291 pairs were matched and extracted ( Fig. 1 ). Fig. 2 shows the Love plot of the SMD before and after PSM. Of these, 46.9% were male and average withdrawal time was 14.9 minutes. The two groups did not show significant differences in sex, purpose, endoscopist, endoscopy system, colonoscopy, bowel preparation, withdrawal time, or doses of midazolam, pethidine, or propofol after PSM ( Table 1 ). As shown in Table 2 , ADR in patients aged 75 to 79 years was significantly higher than that in patients aged 70 to 74 years (66.5% vs 62.2%, *P* = 0.021). APC in patients aged 75 to 79 years was larger than that in patients aged 70 to 74 years (1.54 vs 1.38, *P* = 0.014). There were no significant differences between the two groups in terms of SSLDR (4.5% vs 5.6%) or SSLPC (0.05 vs 0.07). The two groups did not show significant differences in respiratory depression (2.6% vs 2.3%), hypotension (0.8% vs 1.0%), or DPPB (0.2% vs 0.4%). One patient aged 70 to 74 years was hospitalized because of arrhythmia after colonoscopy. There were no cases with perforation or uncontrollable immediate bleeding.

**Table:** **Table 1** Baseline characteristics before and after propensity-score matching.

	Before matching				After matching			
	70 to 74 yr	75 to 79 yr	*P* value	SMD	70 to 74 yr	75 to 79 yr	*P* value	SMD
n	2079	1323			1291	1291		
Male sex, %	49.0	46.3	0.089	0.060	47.1	46.8	0.875	0.006
Indication (A/B/C) ^*^	259/689/1131	196/384/743	0.890	0.008	158/416/717	192/375/724	0.459	0.029
Endoscopist (Expert/Standard)	1469/610	1045/278	< 0.001	0.193	1014/277	1015/276	0.962	0.002
Endoscopy system (X1/ELITE)	1104/975	745/578	0.065	0.064	713/578	714/577	0.968	0.002
Colonoscope (A/B/C) ^†^	310/1667/102	173/1067/83	0.041	0.074	173/1040/78	171/1049/71	0.819	0.009
Bowel preparation (A/B/C) ^‡^	641/1115/323	343/726/254	< 0.001	0.129	330/738/223	340/714/237	0.905	0.005
Withdrawal time, min ± SD	14.7 +- 4.5	15.0 ± 4.7	0.034	0.074	14.9 ± 4.5	14.9 ± 4.7	0.986	0.001
Midazolam, mg ± SD	2.3 ± 1.3	2.3 ± 1.0	0.651	0.040	2.4 ± 1.3	2.3 ± 1.1	0.696	0.015
Pethidine, mg ± SD	9.2 ± 10.8	8.5 ± 9.8	0.332	0.060	8.4 ± 10.2	8.6 ± 9.8	0.630	0.018
Propofol, mg ± SD	5.0 ± 14.0	6.2 ± 15.8	0.015	0.079	5.7 ± 15.4	5.7 ± 15.0	0.938	0.003
SMD, standardized mean difference. *Indications: A, evaluation of symptoms; B, screening; C, surveillance. ^†^ Colonoscope (Olympus): A, CF-XZ1200 and CF-EZ1500D; B, CF-HQ290, CF-HQ290Z, CF-H290EC, and PCF-H290Z; C, PCF-PQ260. ^‡^ Bowel preparation: A, good; B, average; C, marginal. *P* values were calculated using Brunner-Munzel and Wilcoxon signed-rank sum tests for before and after matching, respectively. SMD, standardized mean difference.								

**Table:** **Table 2** Comparison between patients aged 70 to 74 and 75 to 79 years.

	70 to 74 yr	75 to 79 yr	Odds ratio	95% CI	*P* value
n	1291	1291			
ADR, %	62.2	66.5	1.21	1.03–1.42	0.021
APC	1.38	1.54	1.06	1.01–1.11	0.014
Advanced ADR,%	3.6	4.0	1.09	0.73–1.63	0.680
Advanced APC	0.039	0.045	1.14	0.80–1.62	0.472
Adenocarcinoma detection rate,%	1.3	2.1	1.60	0.87–2.95	0.132
SSLDR, %	5.6	4.5	0.80	0.56–1.14	0.209
SSLPC	0.069	0.053	0.81	0.62–1.07	0.145
Respiratory depression, %	2.3	2.6	1.12	0.70–1.81	0.629
Hypotension, %	1.0	0.8	0.77	0.34–1.76	0.531
DPPB, %	0.4	0.2	0.60	0.14–2.51	0.484
ADR, adenoma detection rate; APC, mean number of adenomas per colonoscopy; CI, confidence interval; DPPB, delayed post-polypectomy bleeding; SSLDR, sessile serrated lesion detection rate; SSLPC, mean number of sessile serrated lesions per colonoscopy.					

Table 3 shows the subanalysis in which indications for colonoscopy were limited to surveillance of colorectal polyps and the results remained unchanged.

**Table:** **Table 3** Subanalysis of patients for surveillance.

	70 to 74 yr	75 to 79 yr	Odds ratio	95% CI	*P* value
n	722	722			
ADR, %	60.0	67.3	1.37	1.11–1.70	0.004
APC	1.27	1.47	1.09	1.02–1.16	0.015
Advanced ADR,%	1.8	2.4	1.32	0.63–2.73	0.462
Advanced APC	0.019	0.026	1.30	0.68–2.48	0.421
Adenocarcinoma detection rate,%	0.6	0.4	0.75	0.17–3.36	0.706
SSLDR, %	5.5	4.3	0.76	0.47–1.24	0.275
SSLPC	0.068	0.047	0.75	0.50–1.11	0.145
Respiratory depression, %	2.6	2.9	1.09	0.61–1.93	0.770
Hypotension, %	0.8	0.6	0.66	0.19–2.37	0.528
DPPB, %	0.3	0.0	N/A	N/A	N/A
ADR, adenoma detection rate; APC, mean number of adenomas per colonoscopy; CI, confidence interval; DPPB, delayed post-polypectomy bleeding; SSLDR, sessile serrated lesion detection rate; SSLPC, mean number of sessile serrated lesions per colonoscopy.					

Table 4 shows the subanalysis in which indications for colonoscopy were limited to screening and the results remained unchanged.

**Table:** **Table 4** Subanalysis of patients for screening.

	70 to 74 yr	75 to 79 yr	Odds ratio	95% CI	*P* value
n	372	372			
ADR, %	63.2	66.7	1.17	0.86–1.58	0.318
APC	1.57	1.68	1.03	0.96–1.12	0.417
Advanced ADR,%	5.9	7.3	1.25	0.70–2.23	0.461
Advanced APC	0.059	0.083	1.38	0.81–2.33	0.237
Adenocarcinoma detection rate,%	3.0	5.1	1.77	0.83–3.77	0.141
SSLDR, %	3.5	4.6	1.32	0.63–2.76	0.457
SSLPC	0.046	0.059	1.19	0.71–2.02	0.509
Respiratory depression, %	2.2	3.2	1.52	0.61–3.75	0.368
Hypotension, %	1.9	1.6	0.85	0.28–2.57	0.780
DPPB, %	0.3	0.8	3.02	0.31–29.13	0.340
ADR, adenoma detection rate; APC, mean number of adenomas per colonoscopy; CI, confidence interval; DPPB, delayed post-polypectomy bleeding; SSLDR, sessile serrated lesion detection rate; SSLPC, mean number of sessile serrated lesions per colonoscopy.					

Table 5 shows the subanalysis in which indications for colonoscopy were limited to symptoms. There were no significant differences between the two groups.

**Table:** **Table 5** Subanalysis of patients for symptoms.

	70 to 74 yr	75 to 79 yr	Odds ratio	95% CI	*P* value
n	183	183			
ADR, %	65.0	62.8	0.91	0.59–1.39	0.663
APC	1.38	1.48	1.04	0.92–1.18	0.541
Advanced ADR,%	3.8	4.7	1.30	0.47–3.57	0.610
Advanced APC	0.049	0.060	1.16	0.54–2.48	0.700
Adenocarcinoma detection rate,%	2.2	2.7	1.26	0.33–4.76	0.736
SSLDR, %	7.1	5.5	0.76	0.33–1.77	0.519
SSLPC	0.077	0.066	0.88	0.43–1.79	0.716
Respiratory depression, %	0.5	0.5	1.00	0.06–16.11	1.000
Hypotension, %	1.1	0.0	N/A	N/A	N/A
DPPB, %	1.1	0.0	N/A	N/A	N/A
ADR, adenoma detection rate; APC, mean number of adenomas per colonoscopy; CI, confidence interval; DPPB, delayed post-polypectomy bleeding; SSLDR, sessile serrated lesion detection rate; SSLPC, mean number of sessile serrated lesions per colonoscopy.					

## Discussion

In this study, patients aged 75 to 79 years showed a higher ADR and APC than those aged 70 to 74 years. In addition, there were no differences in frequencies of respiratory depression and DPPB. Because the ADR and APC were high, it might have been effective in preventing colorectal cancer in patients aged 75 to 79 years as well. If patients aged 75 to 79 years have good activities of daily living (ADL) and are classified as ASA I–II, they would be able to complete colonoscopies safely, similar to those aged 70 to 74 years.

Our clinic has previously reported ADRs of 50.8% in 2021 26 and 55.0% in 2024 in all age groups 29 . In this study, ADRs were 62.0% and 66.4% in patients aged 70 to 74 and 75 to 79 years, respectively. ADRs have been reported to increase with age 30 , and this study indicated that ADRs increased in patients aged 75 to 79 years. Considering the ceiling effect of ADR 31 , further improvement may be difficult.

Considerable evidence suggests that risk associated with colonoscopy increases with age. A population-based cohort study reported adverse gastrointestinal events within 30 days after colonoscopy 32 . Risks were 0.5% for patients aged 66 to 69 years, 0.58% for patients aged 70 to 74 years, 0.72% for patients aged 75 to 79 years, 0.88% for patients aged 80 to 84 years, and 1.21% for patients aged 85 or older years, respectively. Compared with patients aged 66 to 69 years, risk was significantly higher in patients aged 80 years or older. A multicenter study reported complications directly related to colonoscopy within 30 days 33 . Risks were 0.11% for patients aged 40 to 59 years, 0.18% for patients aged 60 to 69 years, 0.35% for patients aged 70 to 79 years, and 0.44% for patients aged 80 or older years, respectively. Incidence of AEs after colonoscopy increases with age.

The United States Preventive Services Task Force determined that screening should not be continued after age 85 years because the risk could exceed the potential benefit 6 . For patients aged 75 to 85 years, the United States guidelines recommend continued routine screening but argue for individualization based on an assessment of benefit, risk, and comorbidities. Assessment of benefits included prior colonoscopy findings and life expectancy. Patients with high-risk adenomas are at higher risk of developing advanced neoplasia than average-risk individuals. Therefore, the potential benefit of surveillance may be higher for average-risk individuals. Although elderly patients with high-risk adenomas may benefit from surveillance, this depends on their life expectancy. Patients aged 75 to 85 years require individualized decisions to continue surveillance.

Life expectancy in Japan is approximately 5 years longer than in the United States. There may also be coordination at the national level. In Japan, screening colonoscopies may target individuals younger than age 80 years. However, comorbidities should be considered. This coordination may be similar in countries with a long life expectancy, such as Japan.

This study had some limitations. First, this is a retrospective single-center study. However, the medical data recordings are well-controlled. Second, timeframes and patient cohorts were limited. Third, cost analysis was not performed. A detailed cost-effectiveness analysis is a topic for future research. Fourth, AEs related to bowel preparation were not analyzed. Fifth, withdrawal time included time required for polypectomy in this study. Both American and European guidelines define appropriate withdrawal time as the duration of mucosal observation, explicitly excluding time spent on polypectomy. Sixth, patients with poor PS were excluded from undergoing colonoscopy based on clinical judgment, and none of these patients were tested after being ruled out during the interview stage.

## Conclusions

In conclusion, colonoscopy in patients aged 75 to 79 years in Japan may be considered safe and effective in carefully selected individuals with good ADL. However, due to the lack of evaluation of certain AEs—especially those related to bowel preparation—these findings should be interpreted with caution. Further studies are warranted to comprehensively assess risks associated with the procedure in this age group.
